# Quantum Monte Carlo study of the phase diagram of solid molecular hydrogen at extreme pressures

**DOI:** 10.1038/ncomms8794

**Published:** 2015-07-28

**Authors:** N. D. Drummond, Bartomeu Monserrat, Jonathan H. Lloyd-Williams, P. López Ríos, Chris J. Pickard, R. J. Needs

**Affiliations:** 1 Department of Physics, Lancaster University, Lancaster LA1 4YB, UK; 2 Theory of Condensed Matter Group, Cavendish Laboratory, University of Cambridge, J. J. Thomson Avenue, Cambridge CB3 0HE, UK; 3 Department of Physics & Astronomy, University College London, Gower Street, London WC1E 6BT, UK

## Abstract

Establishing the phase diagram of hydrogen is a major challenge for experimental and theoretical physics. Experiment alone cannot establish the atomic structure of solid hydrogen at high pressure, because hydrogen scatters X-rays only weakly. Instead, our understanding of the atomic structure is largely based on density functional theory (DFT). By comparing Raman spectra for low-energy structures found in DFT searches with experimental spectra, candidate atomic structures have been identified for each experimentally observed phase. Unfortunately, DFT predicts a metallic structure to be energetically favoured at a broad range of pressures up to 400 GPa, where it is known experimentally that hydrogen is non-metallic. Here we show that more advanced theoretical methods (diffusion quantum Monte Carlo calculations) find the metallic structure to be uncompetitive, and predict a phase diagram in reasonable agreement with experiment. This greatly strengthens the claim that the candidate atomic structures accurately model the experimentally observed phases.

## Abstract (web summary)

Experimental studies of hydrogen at high pressure are challenging, so theory is central to understanding its phase behaviour; however, computed phase diagrams do not agree with previous measurements. Here, the authors use a quantum Monte Carlo method and present results in qualitative agreement with experiment.

## Graphical abstract

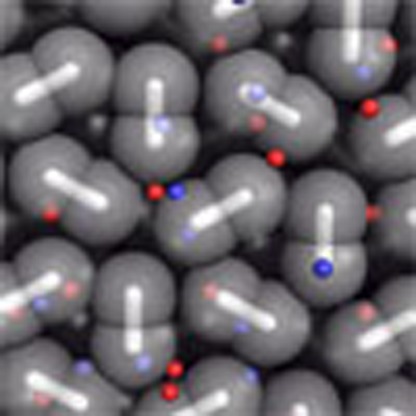

Hydrogen (H) is the simplest and most abundant of all elements and yet it displays amazing richness in its phase behaviour^1^,^2^: it is observed to form a quantum crystalline state and orientationally ordered molecular phases, and it has been predicted to exhibit a liquid-metal phase at high pressures and low temperatures^3^,^4^,^5^, metallic superfluid and superconducting superfluid states^6^,^7^ and high-temperature superconductivity^8^,^9^,^10^. Several crystalline phases of solid molecular H have been observed in diamond anvil cell experiments carried out at pressures up to over 300 GPa (refs 11, 12, 13, 14, 15, 16, 17, 18, 19). The low-pressure phase I, which is a hexagonal close-packed structure formed of freely rotating molecules, transforms to a broken-symmetry phase II, in which the molecular rotations are restricted, at low temperatures^1^,^2^. The transition pressure decreases strongly with isotopic mass^1^,^20^,^21^,^22^,^23^ and also depends on the total spin of the molecules^1^,^22^. As the pressure is increased at low temperatures, there is a further transition from phase II to a phase III at about 160 GPa, with the transition pressure for deuterium (D) exceeding that for H by about 12 GPa (ref. 23). Experimental studies have also demonstrated the existence of a phase IV at temperatures above a few 100 K and pressures above 220 GPa (refs 12, 13, 14, 17, 16). Some constraints on the structures of the observed phases have been obtained from X-ray diffraction experiments^24^,^25^, but the low X-ray scattering cross section of H and the small sample sizes available limit the possible resolution. Infrared (IR) and particularly Raman spectroscopic measurements have yielded valuable information about the vibrational modes of H at high pressures^11^,^12^,^13^,^14^,^15^,^16^,^17^,^18^,^19^,^20^,^21^,^22^,^23^,^24^, but the available experimental data are insufficient to determine the structures of phases II, III and IV.

Candidate structures for phases II, III and IV have been suggested by structure searches based on density functional theory (DFT)^26^,^27^,^28^,^29^,^30^,^31^,^32^, although it should be emphasized that none of these structures has been identified as being unambiguously correct. The candidate structures for phase II consist of packings of molecules with bond lengths almost identical to the zero-pressure value^26^,^27^. We have modelled phase II using a molecular structure of *P*2_1_/*c* symmetry with 24 atoms in the primitive unit cell, which we refer to as *P*2_1_/*c*-24; see Fig. 1a. (We adopt the convention of labelling structures by their symmetry followed by the number of atoms per primitive cell.) *P*2_1_/*c*-24 is the most stable structure found to date in static-lattice DFT within the pressure range appropriate for phase II, and its vibrational characteristics are also compatible with those of phase II. We model phase III using a *C*2/*c*-24 structure consisting of layers of molecules whose bonds lie within the planes of the layers, forming a distorted hexagonal pattern^26^; see Fig. 1b. This very stable structure can account for the high IR activity of phase III^26^. We also consider a molecular *Cmca*-12 structure^26^, which is similar to *C*2/*c*-24, but slightly denser; see Fig. 1c. We model phase IV by a *Pc*-48 structure^28^,^29^, shown in Fig. 1d, which consists of alternate layers of strongly bonded molecules and weakly bonded graphene-like sheets. This type of structure was predicted by Pickard and Needs^26^. *Pc*-48 can account for the occurrence of stiff and soft vibronic modes in phase IV, and its stabilization by temperature. Finally, we consider the *Cmca*-4 structure^33^, which has weaker molecular bonds than *C*2/*c*-24 and *Cmca*-12, and is shown in Fig. 1e. The main goals of our present work are to obtain accurate theoretical results for the relative stabilities of the *P*2_1_/*c*-24, *C*2/*c*-24, *Cmca*-12, *Pc*-48 and *Cmca*-4 structures of H at pressures of 100–400 GPa and temperatures of 0–500 K, and to use these data to construct a temperature–pressure phase diagram of H. We have not considered phase I in our calculations, which is stable at low pressures, because an accurate description of this phase would require a full quantum treatment of the proton spin. Instead we focus our attention on the phase behaviour at higher pressures, where the candidate structures are such that the nuclei are highly localized and hence the motion of the protons is likely to be well-described by collective bosonic vibrational modes.

Useful theoretical descriptions of solid H require very accurate calculations with an energy resolution of a few meV per atom. Various studies have shown that DFT currently cannot provide such accuracy for H structures, as evidenced by the disagreement of results obtained with different exchange-correlation functionals and the fact that DFT predicts H to be metallic at pressures above 300 GPa, in contradiction with experiment^26^,^28^,^29^,^34^,^35^,^36^. We have instead used the diffusion quantum Monte Carlo (DMC) method^37^ to calculate static-lattice energy–volume relations for the different H phases. DMC is generally regarded as the most accurate first-principles method available for carrying out such studies^38^,^39^,^40^. Furthermore, the low mass of the H atom means that a full treatment of quantum nuclear vibrational motion, including anharmonic effects^35^,^40^, is crucial for an accurate description of the energetics. We have therefore used a DFT-based vibrational self-consistent field approach^41^ to calculate anharmonic vibrational energies. We find that the use of DMC (and to a lesser extent the treatment of phonon anharmonicity) renders the metallic *Cmca*-4 structure that is favoured in DFT energetically uncompetitive, leaving us with a phase diagram in reasonable quantitative agreement with experiment.

## Results

### Relative enthalpies

**Figure 2:**
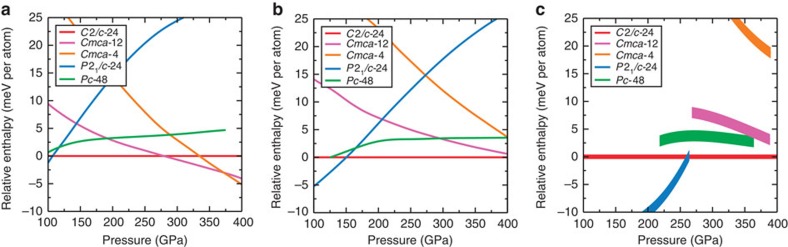
DFT and DMC static-lattice enthalpy–pressure relations for the different H structures relative to *C*2/*c*-24. (**a**) DFT–PBE, (**b**) DFT–BLYP and (**c**) DMC. The relative DFT enthalpies are converged to better than 0.1 meV per atom. The widths of the DMC lines indicate the estimated uncertainties in the enthalpies due to single-particle finite-size errors, which are greater than the uncertainties due to random sampling in the Monte Carlo algorithm, as explained in Supplementary Note 2.

Figure 2 shows the static-lattice enthalpies of the structures relative to *C*2/*c*-24. In Fig. 2a,b we report DFT enthalpies calculated using the Perdew-Burke-Ernzerhof (PBE)^42^ and Becke–Lee–Yang–Parr (BLYP) density functionals^43^,^44^. The relative DFT enthalpies are converged to better than 0.1 meV per atom with respect to **k**-point sampling and plane wave cutoff energy. The difference between the DFT–PBE and DFT–BLYP relative enthalpies arises chiefly from the energetics and not from the slightly different structures obtained from geometry optimization calculations performed at fixed external pressures using the two different functionals: see Supplementary Note 1 and the accompanying Supplementary Fig. 1. In Fig. 2c, we report DMC enthalpies, which were obtained by fitting polynomials to the extrapolated infinite-system-size DMC energies as a function of volume, and differentiating the polynomials to obtain pressures. The structures used for the DMC calculations were obtained from DFT–PBE geometry optimization calculations. We truncate the DMC enthalpy curves at the highest and lowest pressures at which we have performed calculations.

The use of the DMC method has significant consequences for the static-lattice relative enthalpies of the candidate structures. Compared with both DFT–PBE and DFT–BLYP, *Cmca*-4 and *Cmca*-12 are destabilized with respect to *C*2/*c*-24, whereas *P*2_1_/*c*-24 is stabilized with respect to it, but in each case the DFT–BLYP results are closer to the DMC enthalpies, as also found in ref. 36. For *Cmca*-4 and *P*2_1_/*c*-24 the difference between the DMC and the DFT–BLYP results is greater than the difference between the DFT–BLYP and DFT–PBE results, while for *Cmca*-12 these differences are of similar size. Although DFT–BLYP happens to be relatively accurate in the pressure range of interest, it is clear that DFT is unable to provide a consistent, quantitative description of the relative enthalpies of the phases of H.

### Vibrational results

**Figure 3:**
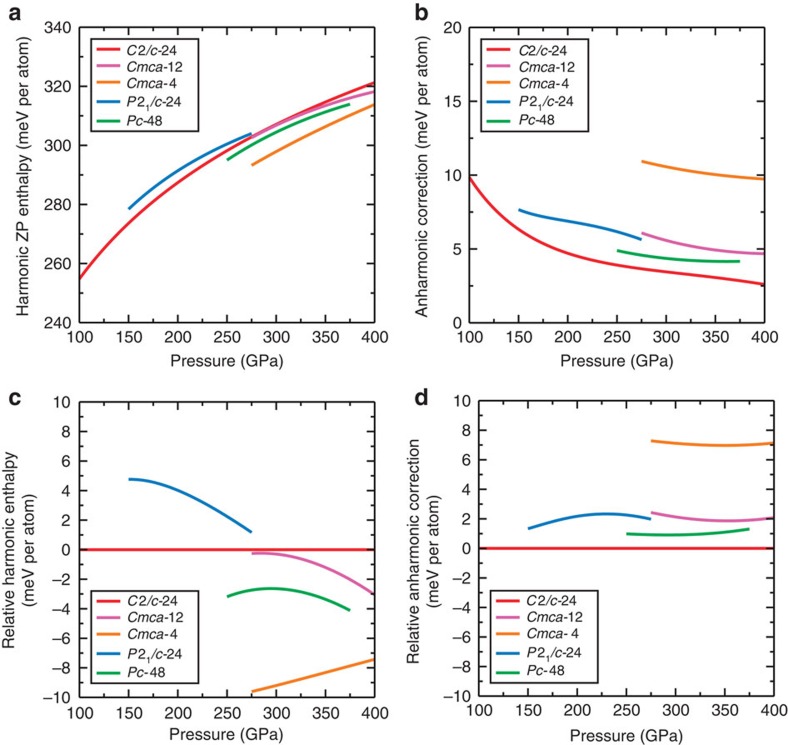
DFT–PBE vibrational contributions to the enthalpies of the H structures. (**a**) Harmonic zero-point (ZP) contributions to enthalpies, (**b**) anharmonic ZP corrections to enthalpies, (**c**) harmonic ZP enthalpies relative to *C*2/*c*-24, and (**d**) anharmonic ZP corrections relative to *C*2/*c*-24. *P*2_1_/*c*-24 is destabilized by both harmonic vibrations and anharmonic corrections, relative to *C*2/*c*-24. *Cmca*-12, *Cmca*-4, and *Pc*-48 are all stabilised by harmonic vibrations but destabilized by anharmonic corrections, relative to *C*2/*c*-24.

The harmonic zero-point contributions to the enthalpies of the H phases increase sublinearly with pressure, as shown in Fig. 3a, while the anharmonic corrections tend to decrease with pressure; see Fig. 3b. The harmonic zero-point enthalpies are roughly 30 times larger than the anharmonic corrections. However, the differences between the harmonic zero-point energies of the five phases considered at fixed pressure are similar in magnitude to the differences between the anharmonic corrections, both being about 10 meV per atom, as shown in Fig. 3c,d. This demonstrates that the variations in the anharmonic vibrational corrections are as important as those of the harmonic contributions to the enthalpies in determining the relative stabilities of phases in this system.

### Structural phase transitions

**Figure 4:**
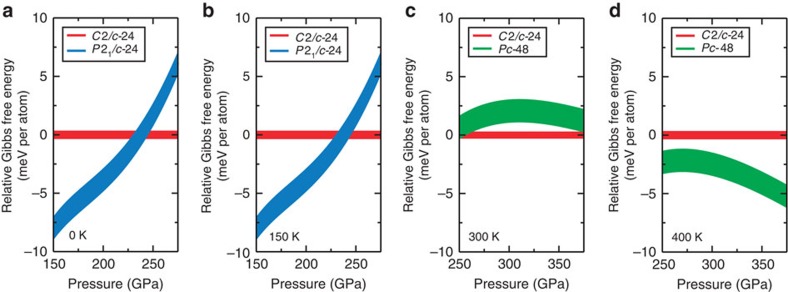
Relative Gibbs free energies of the different H structures. (**a**) 0 K, (**b**) 150 K, (**c**) 300 K and (**d**) 400 K. The Gibbs free energies were calculated using static-lattice DMC calculations together with DFT–PBE harmonic and anharmonic vibrational calculations. The transition from *P*2_1_/*c*-24 to *C*2/*c*-24 occurs at around 235±10 GPa between 0 and 150 K. *Pc*-48 is stabilised by temperature with respect to *C*2/*c*-24. The complete set of relative enthalpies is shown in Supplementary Fig. 2.

**Figure 5:**
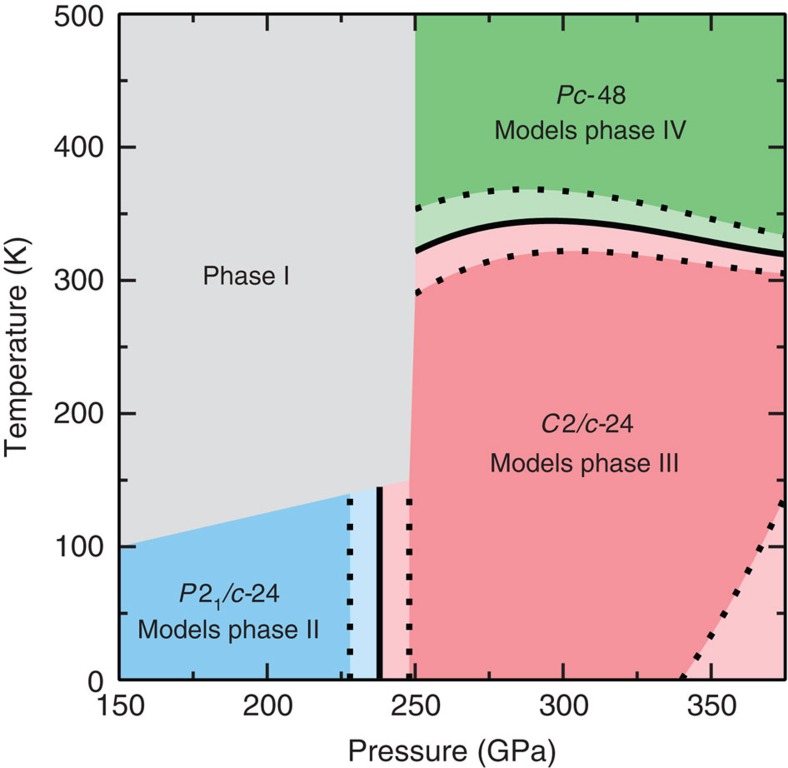
Theoretical temperature–pressure phase diagram for H. The solid black lines show the phase transitions calculated in this work, that is, the set of points at which the relative Gibbs free energy of two phases is zero. The dotted lines show the set of points at which the relative Gibbs free energy is one error bar from zero, and hence indicate the uncertainty in the phase boundaries. At pressures in excess of 350–375 GPa the Gibbs free energies of the *C*2/*c*-24 and *Pc*-48 structures are within error bars of each other. The grey region indicates the temperature–pressure conditions under which phase I is found to exist in experiments.

Figure 4 shows the two structural phase transitions that we have determined in this work, and our theoretical temperature–pressure phase diagram for solid molecular H is shown in Fig. 5. At 0 K, we find a transition from *P*2_1_/*c*-24 to *C*2/*c*-24 at around 235±10 GPa. The corresponding transition pressure for D is 13 GPa higher (note that the difference between H and D is purely due to the DFT vibrational free energy and hence the difference in transition pressures between H and D is relatively precise). Our transition pressure is around 75 GPa greater than those observed experimentally for the transition between phases II and III, but the 13 GPa difference between the transition pressures for H and D agrees well with the experimentally measured value^23^. We note that the theoretical transition pressures between H and D would only differ by around 6 GPa without the inclusion of anharmonic effects.

As shown in Fig. 4, we also find a temperature-driven transition from *C*2/*c*-24 to *Pc*-48 at pressures above 250 GPa and temperatures above 300 K, in good agreement with the experimentally observed transition between phases III and IV. In Fig. 4 we show the relative free energies of *C*2/*c*-24 and *Pc*-48 at 300 and 400 K. At the lower temperature, *C*2/*c*-24 is marginally more stable, but at 400 K, *Pc*-48 has clearly become the more stable structure. The variation in the transition temperature with pressure is smaller than the uncertainty in that quantity, and so we report the *C*2/*c*–24 to *Pc*–48 transition temperature as 320±20 K.

## Discussion

Our theoretical H phase diagram is in reasonable quantitative agreement with experiment, indicating that the *P*2_1_/*c*-24, *C*2/*c*-24, and *Pc*-48 structures provide satisfactory models for phases II, III, and IV. These model structures reproduce the experimental Raman and IR spectra quite well. However, there is a significant disagreement of about 75 GPa between the experimental and theoretical phase II–III transition pressure at 0 K. There are several possible reasons for our substantially larger phase II–III transition pressure. First, the actual structure of phase III may be more stable than the *C*2/*c*-24 model structure. However, *C*2/*c*-24 is the most stable non-metallic structure found in DFT searches over a wide range of pressures, and it is compatible with the Raman and infrared spectra of the observed phase III. If a significantly more stable structure than *C*2/*c*-24 were to be found for phase III, the excellent description of the transition from phase III to IV with increasing temperature obtained with our calculated data would be spoilt. Another possible explanation for the discrepancy with experiment regarding the phase II–III transition pressure could be that we have neglected a significant contribution to the energy of *P*2_1_/*c*-24 (our model for phase II). In particular, our calculations do not account for nuclear exchange effects, which are known to have a significant effect on the phase I-II transition pressure^22^. However, reliable estimates of the size of nuclear exchange effects in solid H at high pressure are not currently available. Furthermore, nuclear exchange effects are expected to be much smaller in D than H, because deuterons are bosons, whereas protons are fermions, and each deuteron has twice the mass of a proton. This suggests that nuclear exchange effects cannot be entirely responsible for the discrepancy in the phase II–III transition pressure in both H and D. Our analysis of different finite-size corrections in Supplementary Note 2 (see also the accompanying Supplementary Figs 3 and 4) indicates that finite-size effects in our relative enthalpies are well-controlled, but it is always possible that finite-size effects may be larger than anticipated. Finally, the fixed-node approximation is an uncontrolled source of error in our DMC calculations and, although fixed-node errors should largely cancel when relative energies are calculated, it cannot be ruled out that fixed-node errors may be larger in one phase than another.

The results we obtain by combining our DMC static-lattice energies and harmonic and anharmonic vibrational energies resolve a discrepancy between DFT and experiment for the transition between phases III and IV. The *Pc*-48 structure was proposed as a candidate for phase IV in refs 28, 29 because its Raman spectrum agrees well with the experimental one and because its weakly bonded layers lead to soft vibrational modes that thermally stabilize it. However, DFT static-lattice calculations together with the harmonic approximation for nuclear motion (used in refs 28, 29) predict the *Cmca*-4 structure to be energetically favoured at all temperatures in the relevant pressure range (note that *Cmca*-4 is stabilised significantly by harmonic zero-point energy; at the static-lattice DFT level it is not competitive, as shown in Fig. 2). The metallic nature of *Cmca*-4 contradicts experiment, in which insulating structures containing strong molecular bonds are found up to pressures in excess of 300 GPa. The phase diagram predicted by DFT is shown in Supplementary Fig. 5. The use of static-lattice DMC energies and anharmonic vibrational energies destabilizes *Cmca*-4, and we find that it is thermodynamically unstable over the entire pressure and temperature range considered here. Our results establish that DFT does not provide even a qualitatively correct description of the phase behaviour of hydrogen. We also find that *Cmca*-12 is unstable at the pressures and temperatures studied in this work. We have found an important discrepancy between our calculated phase II–III transition pressure and experiment, which is currently unresolved, although we have described possible physical reasons for the disagreement. Our calculations demonstrate that anharmonic vibrational effects are crucial for determining the relative stabilities of the phases.

## Methods

### Quantum Monte Carlo calculations

The DMC method^37^,^45^ is capable of delivering much higher accuracy than DFT, and the scaling of the computational cost with system size enables the simulation of the hundreds of atoms required for accurate calculations. We have used the DMC method to calculate static-lattice energies using H structures relaxed within DFT–PBE at a given external pressure. In DMC, the ground-state component of a trial wave function is projected out by simulating the Schrödinger equation in imaginary time, subject to the constraint that the nodal surface of the wave function is fixed to be that of the trial wave function^37^,^45^. We used Slater–Jastrow wave functions as implemented in the CASINO code^46^. Full technical details of our calculations can be found in Supplementary Note 2. The single-particle orbitals were obtained from the CASTEP code^47^ using the PBE exchange-correlation functional. The nuclei were represented by bare Coulomb potentials and appropriate cusp corrections were applied to the orbitals. We used a flexible Jastrow factor^48^ whose parameters were optimized using variational Monte Carlo^49^. Variational Monte Carlo and DMC simulations were performed using 96 and 768 atoms, and the results were extrapolated to infinite system size. Using the resources of the Oak Ridge Leadership Computing Facility, we achieved statistical error bars of <0.3 meV per atom in all our DMC calculations.

### Anharmonic vibrational calculations

We have calculated harmonic vibrational free energies by using the finite-displacement method to construct the matrix of force constants and diagonalising the corresponding dynamical matrices over a fine vibrational Brillouin-zone grid, as described in Supplementary Note 3 (with accompanying data presented in Supplementary Figs 6 and 7). We determined anharmonic corrections to the harmonic free energies using a vibrational self-consistent field method^40^,^41^,^50^, sampling the low-energy part of the DFT–PBE Born–Oppenheimer energy surface along harmonic normal modes to large amplitudes. The resulting anharmonic Schrödinger equation for the nuclear motion was solved by expanding the wave function in a basis of simple harmonic oscillator eigenstates. Thermal occupation of excited states allowed us to calculate free energies at arbitrary temperatures. The vibrational free energy differences between the structures were converged to better than 1 meV per atom. Our approach does not describe possible melting.

### Data availability

All relevant data present in this publication can be accessed at: http://www.repository.cam.ac.uk/handle/1810/248864.

## Additional information

**How to cite this article:** Drummond, N. D. *et al.* Quantum Monte Carlo study of the phase diagram of solid molecular hydrogen at extreme pressures. *Nat. Commun.* 6:7794 doi: 10.1038/ncomms8794 (2015).

## Supplementary Material

Supplementary Information Supplementary Figures 1-7, Supplementary Notes 1-3 and Supplementary References

## Figures and Tables

**Figure 1:**
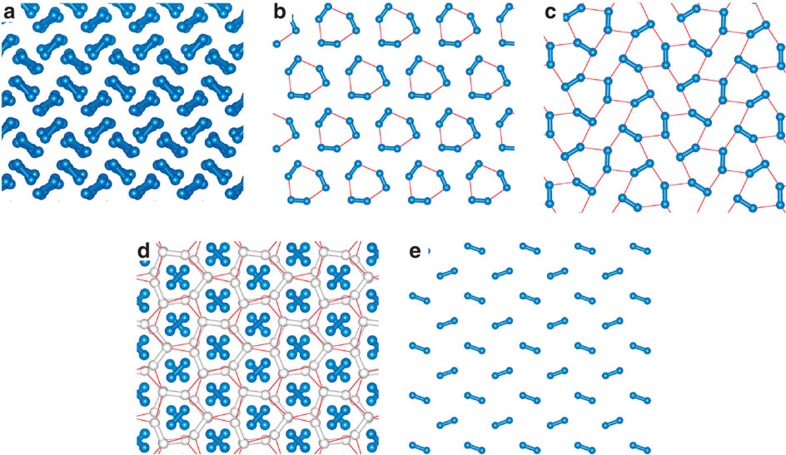
Atomic structures of the five H phases considered in this work. (**a**) *P*2_1_/*c*-24, (**b**) *C*2/*c*-24, (**c**) *Cmca*-12, (**d**) *Pc*-48 and (**e**) *Cmca*-4. The blue dumbbells indicate short bonds between atoms (<0.8 Å). The white dumbbells indicate long bonds between atoms (< 0.9 Å). The red lines indicate close contacts between atoms (<1.2 Å) in the layered structures. *P*2_1_/*c*-24 consists of molecules arranged on a distorted hexagonal close-packed lattice. *C*2/*c*-24, *Cmca*-12 and *Cmca*-4 consist of layers of molecules whose bonds lie within the planes of the layers, forming distorted hexagonal patterns, and we show top–down views of single layers. *Pc*-48 consists of alternate layers of isolated strongly bonded molecules and weakly bonded graphene-like sheets, and we show a top–down view of four layers. The structures are shown at a common DFT–PBE pressure (250 GPa).
