# Supplementary material for: Quantum Monte Carlo study of the phase diagram of solid molecular hydrogen at extreme pressures
Source: Nat Commun. 2015 Jul 28;6:7794. doi: 10.1038/ncomms8794 (PMC4525154; doi:10.1038/ncomms8794)
Supplement: Supplementary Information — Supplementary Figures 1-7, Supplementary Notes 1-3 and Supplementary References [file ncomms8794-s1.pdf]

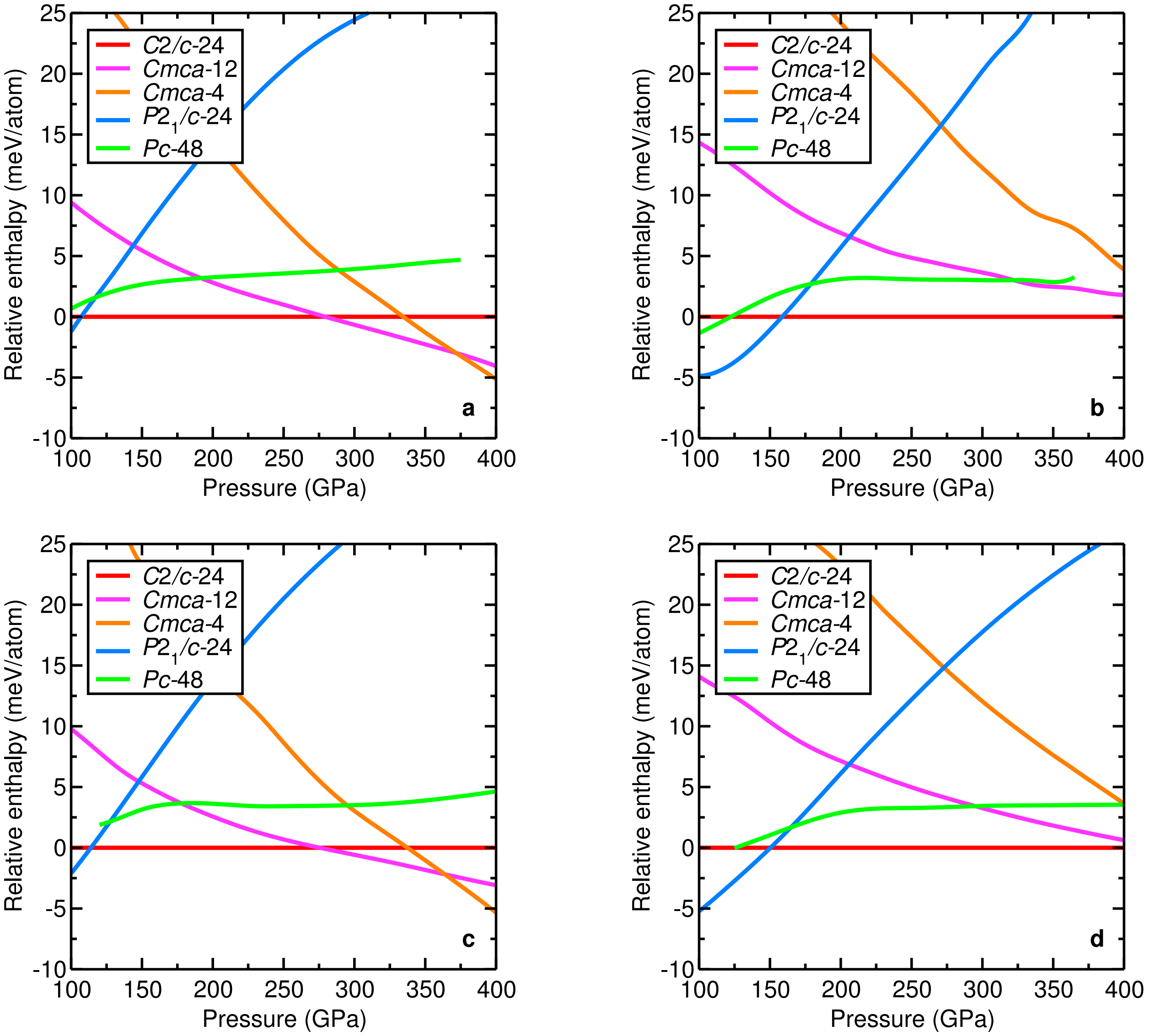

Supplementary Figure 1: DFT-PBE and DFT-BLYP enthalpy and geometry comparison. Results are shown for (**a**) DFT-PBE geometries and DFT-PBE static-lattice enthalpies, (**b**) DFT-PBE geometries and DFT-BLYP static-lattice enthalpies, (**c**) DFT-BLYP geometries and DFT-PBE static-lattice enthalpies, and (**d**) DFT-BLYP geometries and DFT-BLYP static-lattice enthalpies.

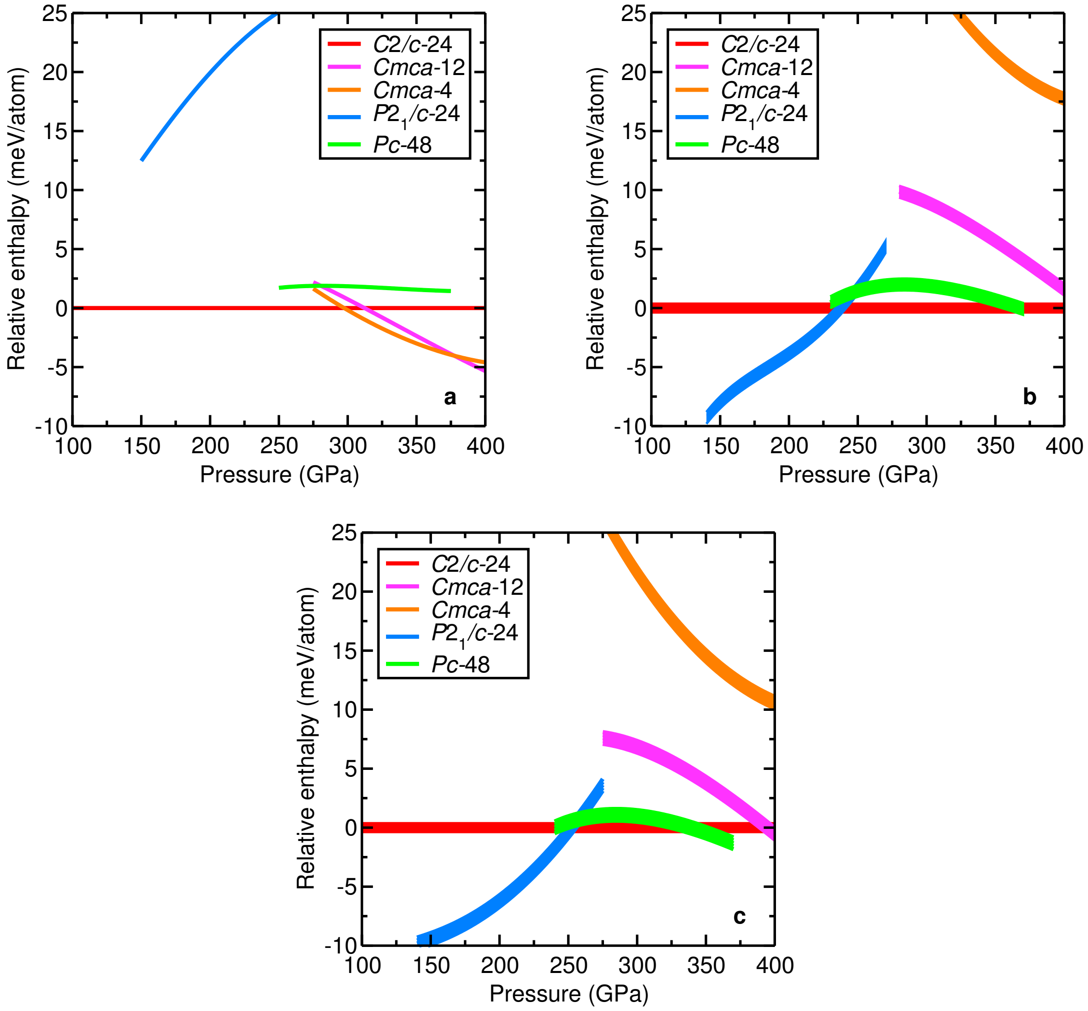

Supplementary Figure 2: Zero-temperature enthalpies for all phases relative to *C*2/*c*-24 as calculated using (a) DFT-PBE with harmonic and anharmonic DFT-PBE vibrational corrections, (b) DMC with harmonic and anharmonic DFT-PBE vibrational corrections and (c) DMC with harmonic (and no anharmonic) DFT-PBE vibrational corrections. It is clear that the *Cmca*-4 structure, which is the most stable structure in DFT calculations over a broad pressure range[1], is uncompetitive in our DMC calculations. The only transition that is significantly affected by temperature is the one from the *C*2/*c*-24 structure to *Pc*-48, as explained in the main body of our article. Comparing panels (**a**), (**b**), and (**c**), it can be seen that our revision of the theoretical phase diagram of solid hydrogen is primarily due to our use of DMC rather than our use of anharmonic vibrational corrections.

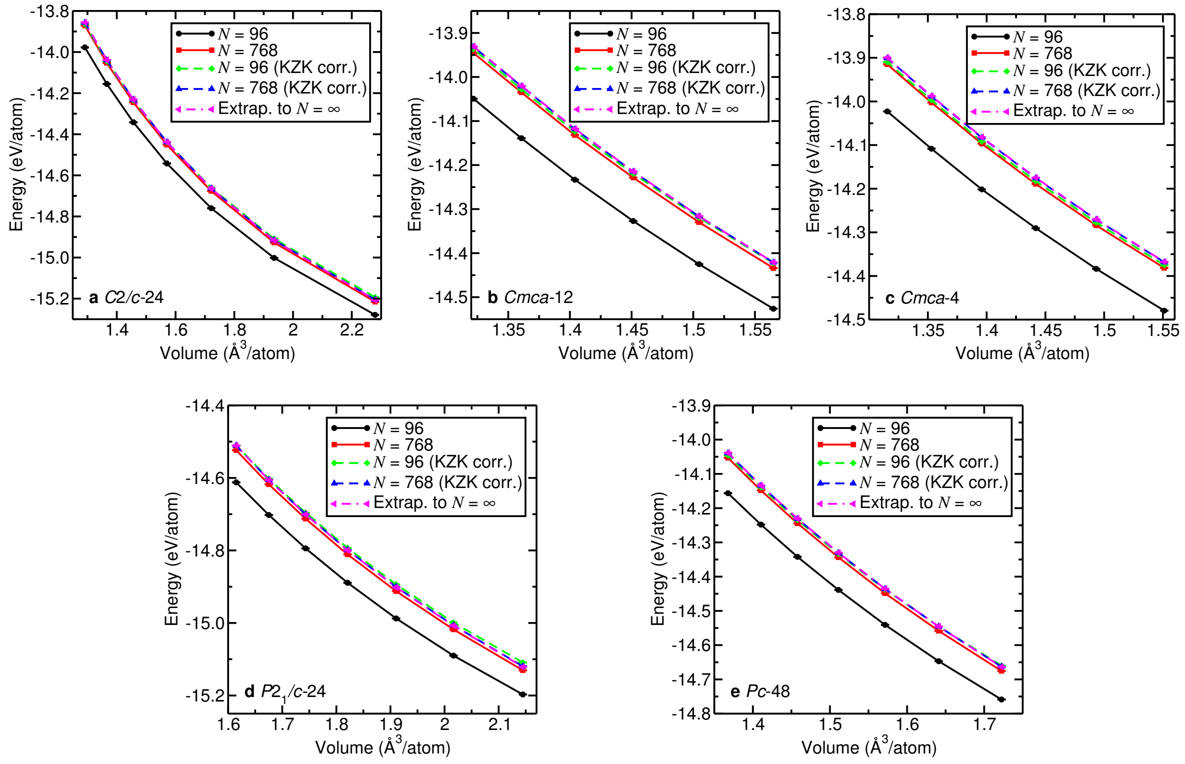

Supplementary Figure 3: Static-lattice DMC energy against volume. Results are shown for the (**a**) $C2/c$-24, (**b**) *Cmca*-12, (**c**) *Cmca*-4, (**d**) $P2_1/c$-24, and (**e**) *Pc*-48 structures of H. Twist-averaged results obtained at finite system sizes N together with data corrected using the Kwee-Zhang-Krakauer scheme[2] and data extrapolated to the thermodynamic limit are shown.

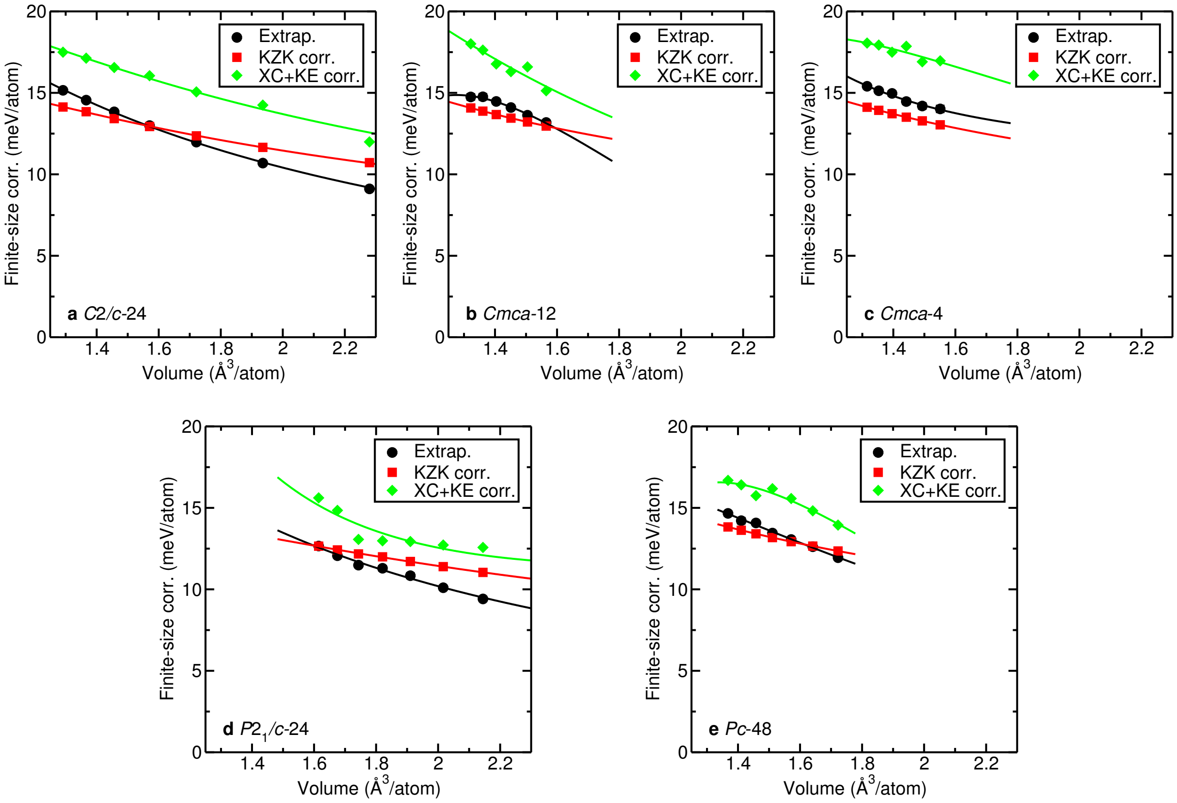

Supplementary Figure 4: Finite-size corrections to the energy per atom in a 768-atom cell against volume. Results are shown for the (**a**) *C*2/*c*-24, (**b**) *Cmca*-12, (**c**) *Cmca*-4, (**d**) $P2_1/c$-24, and (**e**) *Pc*-48 structures of H. The three corrections shown are the results of extrapolation to infinite system size by fitting Supplementary Eq. (2) to the energy data at $N = 96$ and $N = 768$ minus the energy at $N = 768$; the Kwee-Zhang-Krakauer DFT-based correction[2]; and the sum of the exchange-correlation and kinetic-energy corrections proposed in Supplementary Refs. 3 and 4. The solid lines are fits of quadratics in Ω^{-1} to the corrections, where Ω is the volume per atom.

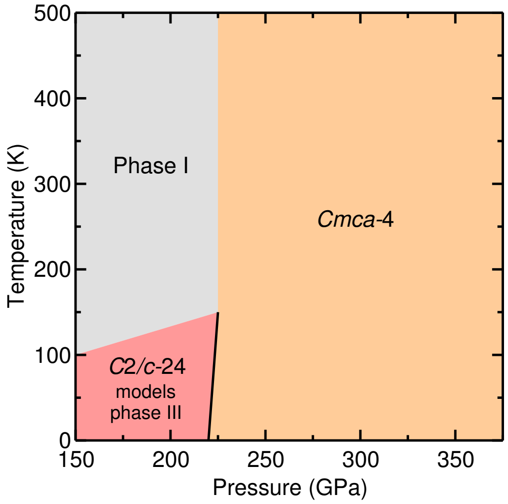

Supplementary Figure 5: Phase diagram predicted by DFT-PBE calculations (with harmonic-phonon free energies). The DFT-PBE phase diagram is strikingly different from the DMC phase diagram reported in the main body of our paper: at high pressure it is dominated by the metallic *Cmca*-4 phase, which is energetically irrelevant in our DMC calculations and does not match the experimentally observed insulating behaviour of hydrogen at these pressures. It should be noted that the DFT-PBE phase diagram presented in Supplementary Ref. 5 is incorrect; the DFT-PBE phase diagram shown here is in agreement with an erratum to that paper[1].

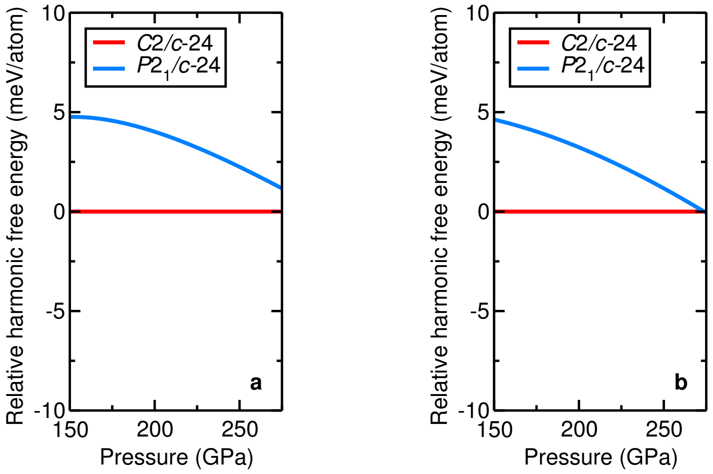

Supplementary Figure 6: Dependence of harmonic contributions to relative Gibbs free energies on density functional used for vibrational calculations at 0 K. The harmonic vibrational free energies were calculated using (**a**) DFT-PBE and (**b**) DFT-BLYP.

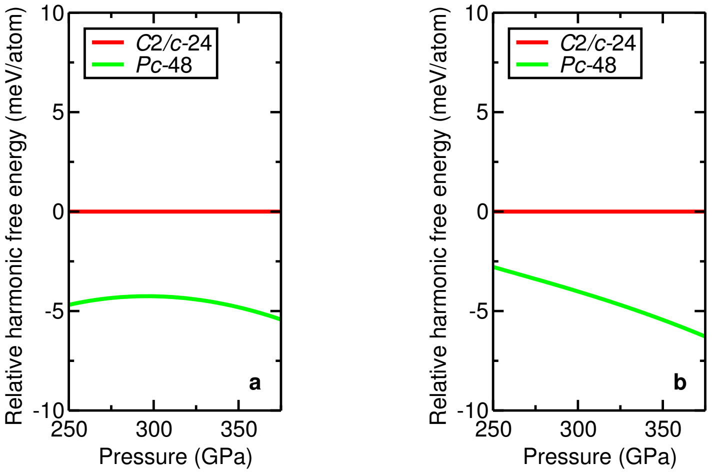

Supplementary Figure 7: Dependence of harmonic contributions to relative Gibbs free energies on density functional used for vibrational calculations at 300 K. The harmonic vibrational free energies were calculated using (**a**) DFT-PBE and (**b**) DFT-BLYP.

Supplementary Note 1. Geometry optimisation

We have studied the $P2_1/c$-24, $C2/c$-24, *Cmca*-12, *Pc*-48, and *Cmca*-4 structures of hydrogen at a range of pressures suggested by previous theoretical work on the phase diagram[1,5–8]. The structures are described and motivated in the main body of our article. The geometries used in our quantum Monte Carlo calculations were determined by relaxing the lattice vectors and internal parameters of each phase within DFT at fixed external pressures. The geometry optimisation calculations were performed using the CASTEP plane-wave basis code[9], the Perdew-Burke-Ernzerhof (PBE) generalised gradient approximation density functional[10], and an ultrasoft pseudopotential[11]. We used a plane-wave cutoff energy of 1200 eV and a Monkhorst-Pack[12] Brillouin zone sampling grid of spacing $2\pi \times 0.02$ Å^{-1}. The forces on the atoms were converged to less than 0.01 eV/Å and the components of the stress tensor were converged to less than 0.01 GPa. We tested the dependence of the relative enthalpies as a function of pressure on the density functional used to perform the geometry optimisation calculations. The DFT-PBE static-lattice relative enthalpies with DFT-PBE and DFT-BLYP geometries shown in Supplementary Figs. 1(a) and 1(c) are very similar, as are the DFT-BLYP static-lattice relative enthalpies with DFT-PBE and DFT-BLYP geometries shown in Supplementary Figs. 1(b) and 1(d). Changing the functional used to optimise the geometry alters the relative enthalpies by about 1 meV/atom.

We have directly verified that our DMC calculation of the transition pressure between the $P2_1/c$-24 and $C2/c$-24 structures (which model phases II and III, respectively) is robust against the choice of method used to optimise the geometry. If the DFT-PBE geometry is replaced by the DFT-BLYP geometry then the DMC enthalpies of 96-atom cells of the $P2_1/c$-24 and $C2/c$-24 structures are both reduced by 10.0(4) meV/atom; however the estimated transition pressure between the structures is reduced by an insignificant amount [5(7) GPa]. The fact that the DMC enthalpies with DFT-BLYP geometries are lower than the DMC enthalpies with DFT-PBE geometries indicates that the DFT-BLYP geometries are more accurate, as also reported in Supplementary Ref. 13. With hindsight it would have been preferable to use DFT-BLYP geometries in all our calculations; nevertheless, we have at least verified that our choice of geometry-optimisation method

has negligible effect on the relative enthalpies and hence predicted phase boundaries.

Supplementary Note 2. Quantum Monte Carlo calculations

All our quantum Monte Carlo (QMC) calculations were performed using the CASINO code[14]. Our trial wave functions consisted of products of Slater determinants of single-electron orbitals multiplied by symmetric Jastrow correlation factors $\exp(J)$. The Jastrow exponents J consisted of polynomial and plane-wave expansions in the electron-electron distances, polynomial expansions in the electron-nucleus distances, and polynomial terms coupling the electron-electron and electron-nucleus separations[15]. The polynomial electron-electron term was smoothly cut off at the radius of the largest sphere that can be inscribed in the Wigner-Seitz cell of the simulation cell. The electron-nucleus and electron-electron-nucleus terms were smoothly truncated at optimisable cutoff lengths. A total of 112 optimisable parameters were present in the Jastrow exponent, including the free cutoff lengths. The free parameters in J were optimised by minimising the variational Monte Carlo (VMC) energy expectation value[16], with the exception of the cutoff lengths, which were optimised by minimising the mean absolute deviation of the local energies of VMC-sampled sets of configurations from the median local energy. The orbitals in the Slater determinants were taken from DFT calculations using the CASTEP code[9] and the PBE density functional[10]. The nuclei were represented by bare Coulomb potentials. A plane-wave cutoff energy of 5442.28 eV ($= 200$ Ha) was used in our DFT wave-function-generation calculations. The plane-wave orbitals were re-represented in a B-spline (blip) basis[17] to improve the scaling of the QMC calculations with system size, and the orbitals were modified in the vicinity of the nuclei to impose the electron-nucleus Kato cusp conditions[18,19].

Our DMC calculations were performed at two different time steps, 0.01 and 0.04 a.u., with the target configuration population being four times larger in the former case. We extrapolated our energies linearly to zero time step. This simultaneously extrapolated the DMC energy to infinite population, because time-step bias is linear in the time step and population-control bias falls off as the reciprocal of the target population. Coupling between time-step and finite-population biases is proportional to the product of the time step and the inverse population, and can therefore be assumed to be negligible; numerical tests for the homogeneous electron gas confirm that time-step

and finite-population biases are essentially independent of one another.

Through the use of the enormous resources of Oak Ridge Leadership Computing Facility, we were able to achieve statistical error bars of less than 0.3 meV/atom in all our DMC calculations.

The dominant sources of error in QMC studies of condensed matter are usually finite-size effects. Finite-size errors in the total energy primarily consist of (i) oscillatory single-particle finite-size effects due to momentum quantisation (analogous to $\mathbf{k}$-point sampling errors in DFT) and (ii) systematic effects due to the long range of both the Coulomb interaction and two-body correlations. Single-particle errors may be greatly reduced by twist averaging in the canonical ensemble[20] (i.e., averaging the DMC energy over offsets $\mathbf{k}_\mathrm{s}$ to the grid of $\mathbf{k}$ vectors at a given system size), while long-range effects can be reduced by extrapolation or corrections[2–4]. We have carried out twist-averaged DMC calculations at two different system sizes ($N = 96$ and $N = 768$ atoms) for each phase and volume. To reduce finite-size effects further, for each structure and system size we chose our supercell lattice vectors to be such that the radius of the largest sphere that can be inscribed in the supercell is maximised (and hence the distance between the nearest images of each particle is made as large as possible). To perform the twist averaging, we fitted

$$E(N, \mathbf{k}_\mathrm{s}) = \bar{E}(N) + b\,[E_\mathrm{PBE}(N, \mathbf{k}_\mathrm{s}) - E_\mathrm{PBE}(\infty)] \tag{1}$$

to the DMC energies $E(N, \mathbf{k}_\mathrm{s})$ obtained with 24 randomly chosen $\mathbf{k}$-vector offsets $\mathbf{k}_\mathrm{s}$, where $\bar{E}(N)$ is the twist-averaged energy in the N-atom cell and b is a fitting parameter. $E_\mathrm{PBE}(N, \mathbf{k}_\mathrm{s})$ is the DFT-PBE energy obtained with exactly the same grid of $\mathbf{k}$ vectors as the corresponding DMC calculation, while $E_\mathrm{PBE}(\infty)$ is the DFT-PBE energy obtained with a fine $\mathbf{k}$-point grid, in which the maximum spacing of $\mathbf{k}$ points was 0.0189 Å^{-1}. The Jastrow factor was optimised within VMC once for each system size N, using a value of $\mathbf{k}_\mathrm{s}$ for which the system had time-reversal symmetry. We tested each possible such $\mathbf{k}_\mathrm{s}$ to find the one for which the DFT energy was closest to the DFT energy with a fine $\mathbf{k}$-point mesh, and used this $\mathbf{k}_\mathrm{s}$ in our optimisation calculations.

The twist-averaging process introduces an uncertainty of due to the finite number of random $\mathbf{k}_\mathrm{s}$ values used and in our calculations the magnitude of this uncertainty is greater than that inherent

in the DMC method due to the finite number of electronic configurations visited during the random walk. Performing a least-squares fit of the twist-averaged energy-volume data to an analytic function also has an associated uncertainty. These two sources of uncertainty are not independent, since we use the same set of twist angles in fractional coordinates for each structure at different volumes, making the energy difference between any two points in the same curve more precise than the uncertainty in the absolute energy of any of the individual points would suggest. The effect of residual single-particle finite-size errors therefore reduces to an uncertainty in the vertical shift of the energy-volume and enthalpy-pressure curves without affecting their shape.

We considered three entirely different approaches to correct long-range finite-size errors. The first approach is to extrapolate the twist-averaged energy per atom to the thermodynamic limit by fitting

$$\bar{E}(N) = E(\infty) + cN^{-1} \tag{2}$$

to our DMC data at the two different system sizes $N = 96$ and $N = 768$, where c is a fitting parameter and $E(\infty)$ is the energy per atom in the infinite-system limit. This fitting form is appropriate[3], because the finite-size error in the energy per atom falls off asymptotically as N^{-1}. The second approach, proposed by Kwee, Zhang, and Krakauer (KZK)[2], is to add a correction given by the difference of the DFT energy in the usual local density approximation (LDA) minus the DFT energy obtained with a modified LDA functional appropriate for a finite homogeneous electron gas contained in the simulation-cell volume. The KZK DFT-LDA calculations were performed with a fine $\mathbf{k}$-point sampling, because the correction was applied to twist-averaged DMC data. As can be seen in Supplementary Fig. 3, the two methods for removing finite-size effects are in good agreement; in particular the KZK-corrected DMC data with $N = 768$ atoms are very close to the results obtained by extrapolation using Supplementary Eq. (2). Supplementary Fig. 4 shows the finite-size corrections to the energy data with $N = 768$ atoms in greater detail.

A third approach for removing long-range finite-size errors is that introduced by Chiesa *et al.*[3] and further developed by Drummond *et al.*[4], in which corrections based on the static structure factor and the two-body Jastrow factor are applied to remove finite-size errors in the exchange-

correlation energy and the kinetic energy, respectively. We find that the sum of the exchange-correlation and kinetic-energy corrections for the hydrogen structures with $N = 768$ atoms is systematically higher than the correction corresponding to extrapolation to infinite system size by about 3 meV/atom, as shown in Supplementary Fig. 4. The exchange-correlation and kinetic-energy corrections suffer from random errors due to the fact that they make use of stochastically optimised wave functions that were generated at one particular $\mathbf{k}_s$ value for each phase and pressure. In particular, the correction to the kinetic energy relies on the fitting of a model electron-electron Jastrow term $u(k)$ to two-body Jastrow terms optimised in VMC calculations. Furthermore, the static structure factor that we used to evaluate the exchange-correlation correction was obtained from DMC data; an extrapolated estimate[21] of the static structure factor using VMC and DMC data would be more accurate. The KZK approach does not rely on a stochastically optimised wave function, and gives noise-free relative energies. However, it is clear from Supplementary Fig. 4 that, whichever correction is applied, the finite-size errors in the relative energies are much smaller than 3 meV per atom.

Our final results for the energies in the limit of infinite system size were obtained by extrapolation using our DMC energy data at $N = 96$ and $N = 768$. Extrapolation has the advantage that it can approximately account for finite-size effects that are not considered in the correction schemes, such as finite-size effects in the fixed-node error. Furthermore, it does not suffer from the reliance on stochastically optimised trial wave functions that affects the kinetic-energy correction, because it is purely based on Slater-Jastrow DMC energies.

We have performed DMC calculations using Slater-Jastrow-backflow trial wave functions[22,23] for systems with $N = 96$ atoms. Unlike a Jastrow factor, a backflow function modifies the nodal surface of the Slater wave function and hence can potentially improve the fixed-node[24] DMC energy. Unfortunately, we find that the difference between the Slater-Jastrow and Slater-Jastrow-backflow DMC energies exhibits a noisy dependence on both the system size and the choice of simulation-cell Bloch vector $\mathbf{k}_s$ used for the optimisation of the backflow function. We therefore report only DMC results obtained using Slater-Jastrow wave functions (for which the DMC energy

is independent of the Jastrow factor and hence the Bloch vector $\mathbf{k}_s$ at which it was optimised).

In order to find enthalpy–pressure curves for the different phases, we fitted model equations of state $E(\Omega)$ to our finite-size-corrected DMC energy per atom against volume per atom Ω. From these models it is straightforward to determine the pressure $p = -(\partial E/\partial\Omega)$ at zero temperature and hence obtain the enthalpy per atom $H = E + p\Omega$. We investigated several choices of model, including the Vinet equation of state[25], polynomials, and cubic spline fits to our energy-volume data. For *Cmca*-12, *Cmca*-4, $P2_1/c$-24, and *Pc*-48, we found that a quadratic in Ω^{-1} gives a relatively good fit to our DMC $E(\Omega)$ data with lower χ^2 values than the Vinet equation of state. For *C*2/*c*-24, we used a cubic polynomial in Ω^{-1}. We found that cubic splines tend to overfit our data.

An alternative approach for determining the pressure directly from the QMC calculations would be to use the virial theorem. However, the resulting estimate suffers from much larger noise than the total energy (there is no zero-variance theorem for the pressure) and furthermore the virial estimate of the pressure depends on the entire trial wave function, not just its nodal surface. We find that it is much more precise and accurate to determine the pressure by fitting model equations of state to energy-volume data.

Supplementary Note 3. Lattice dynamics

We have performed quasiharmonic calculations to evaluate the harmonic vibrational contribution to the free energy at a range of pressures. We constructed the matrix of force constants using the finite-displacement method[26] by averaging over positive and negative displacements of a magnitude of 0.005 Å. We then transformed the real-space force-constant matrix to the reciprocal-space dynamical matrix and diagonalised the latter to obtain the harmonic frequencies $\omega_n(\mathbf{k})$ over a Fourier-interpolated grid of $\mathbf{k}$-space points and branches n. All our harmonic free energies are converged to better than 0.1 meV per atom, which required simulation cells containing 192 atoms for *P*$2_1/c$-24 and *C*2/*c*-24, 108 atoms for *Cmca*-4, and 96 atoms for *Cmca*-12 and *Pc*-48.

We have also performed anharmonic vibrational calculations using the method described in Supplementary Ref. 27. We mapped the Born-Oppenheimer energy surface along the directions determined by the harmonic normal modes, sampling a total of 17 points along each direction and to a maximum amplitude of $5\times\sqrt{\langle q^2_{n\mathbf{k}}\rangle}$, where $q_{n\mathbf{k}}$ is the normal-mode coordinate of the vibrational mode $(n,\mathbf{k})$. We then solved the resulting vibrational Schrödinger equation by expanding the vibrational wave function in a basis of simple harmonic oscillator eigenstates determined by a quadratic fit to the mapped Born-Oppenheimer energy surface. Converged results were achieved by including up to 50 basis functions for each degree of freedom.

We have investigated the effect of the choice of density functional used to calculate harmonic vibrational free energies. Calculating vibrational free energies using DFT-BLYP[28,29] instead of DFT-PBE[10] at 0 K has little effect on the harmonic contribution to the relative Gibbs free energies of *C*2/*c*-24 and *P*$2_1/c$-24 (see Supplementary Fig. 6). Calculating vibrational free energies using DFT-BLYP instead of DFT-PBE at 300 K increases the harmonic contribution to the Gibbs free energy of *Pc*-48 relative to *C*2/*c*-24 by about 2 meV per atom at 250 GPa and decreases it by about 1 meV per atom at 400 GPa (see Supplementary Fig. 7). Our final harmonic and anharmonic results were obtained using DFT-PBE.

Supplementary References:

1. Pickard, C. J., Martinez-Canales, M. & Needs, R. J. Erratum: Density functional theory study of phase IV of solid hydrogen [Phys. Rev. B 85, 214114 (2012)]. *Phys. Rev. B* **86**, 059902 (2012).

2. Kwee, H., Zhang, S. & Krakauer, H. Finite-size correction in many-body electronic structure calculations. *Phys. Rev. Lett.* **100**, 126404 (2008).

3. Chiesa, S., Ceperley, D. M., Martin, R. M. & Holzmann, M. Finite-size error in many-body simulations with long-range interactions. *Phys. Rev. Lett.* **97**, 076404 (2006).

4. Drummond, N. D., Needs, R. J., Sorouri, A. & Foulkes, W. M. C. Finite-size errors in continuum quantum Monte Carlo calculations. *Phys. Rev. B* **78**, 125106 (2008).

5. Pickard, C. J., Martinez-Canales, M. & Needs, R. J. Density functional theory study of phase IV of solid hydrogen. *Phys. Rev. B* **85**, 214114 (2012).

6. Pickard, C. J. & Needs, R. J. Structure of phase III of solid hydrogen. *Nat. Phys.* **3**, 473–476 (2007).

7. Pickard, C. J. & Needs, R. J. Structures at high pressure from random searching. *Physica Status Solidi (b)* **246**, 536–540 (2009).

8. Johnson, K. A. & Ashcroft, N. W. Structure and bandgap closure in dense hydrogen. *Nature* **403**, 632–635 (2000).

9. Clark, S. J. *et al.* First principles methods using CASTEP. *Z. Kristallogr.* **220**, 567–570 (2005). 5/6/2005.

10. Perdew, J. P., Burke, K. & Ernzerhof, M. Generalized gradient approximation made simple. *Phys. Rev. Lett.* **77**, 3865–3868 (1996).

11. Vanderbilt, D. Soft self-consistent pseudopotentials in a generalized eigenvalue formalism. *Phys. Rev. B* **41**, 7892–7895 (1990).

12. Monkhorst, H. J. & Pack, J. D. Special points for Brillouin-zone integrations. *Phys. Rev. B* **13**, 5188–5192 (1976).

13. Clay, R. C. *et al.* Benchmarking exchange–correlation functionals for hydrogen at high pressures using quantum Monte Carlo. *Phys. Rev. B* **89**, 184106 (2014).

14. Needs, R. J., Towler, M. D., Drummond, N. D. & López Ríos, P. Continuum variational and diffusion quantum Monte Carlo calculations. *J. Phys.: Condens. Matter* **22**, 023201 (2010).

15. Drummond, N. D., Towler, M. D. & Needs, R. J. Jastrow correlation factor for atoms, molecules, and solids. *Phys. Rev. B* **70**, 235119 (2004).

16. Umrigar, C. J., Toulouse, J., Filippi, C., Sorella, S. & Hennig, R. G. Alleviation of the fermion-sign problem by optimization of many-body wave functions. *Phys. Rev. Lett.* **98**, 110201 (2007).

17. Alfè, D. & Gillan, M. J. Efficient localized basis set for quantum Monte Carlo calculations on condensed matter. *Phys. Rev. B* **70**, 161101 (2004).

18. Ma, A., Towler, M. D., Drummond, N. D. & Needs, R. J. Scheme for adding electron–nucleus cusps to Gaussian orbitals. *J. Chem. Phys.* **122**, 224322 (2005).

19. Binnie, S. J. *et al.* Bulk and surface energetics of crystalline lithium hydride: Benchmarks from quantum Monte Carlo and quantum chemistry. *Phys. Rev. B* **82**, 165431 (2010).

20. Lin, C., Zong, F. H. & Ceperley, D. M. Twist-averaged boundary conditions in continuum quantum Monte Carlo algorithms. *Phys. Rev. E* **64**, 016702 (2001).

21. Ceperley, D. M. & Kalos, M. H. Quantum many-body problems. In Binder, K. (ed.) *Monte Carlo methods in statistical physics* (Springer-Verlag Heidelberg, 1979).

22. Kwon, Y., Ceperley, D. M. & Martin, R. M. Effects of three-body and backflow correlations in the two-dimensional electron gas. *Phys. Rev. B* **48**, 12037–12046 (1993).

23. López Ríos, P., Ma, A., Drummond, N. D., Towler, M. D. & Needs, R. J. Inhomogeneous backflow transformations in quantum Monte Carlo calculations. *Phys. Rev. E* **74**, 066701 (2006).

24. Anderson, J. B. Quantum chemistry by random walk. H ^2P, H_3^+ D_{3h} $^1A_1'$, H_2 $^3\Sigma_u^+$, H_4 $^1\Sigma_g^+$, Be ^1S. *J. Chem. Phys.* **65**, 4121 (1976).

25. Vinet, P., Ferrante, J., Smith, J. R. & Rose, J. H. A universal equation of state for solids. *J. Phys. C Solid State* **19**, L467–L473 (1986).

26. Kunc, K. & Martin, R. M. *Ab initio* force constants of GaAs: a new approach to calculation of phonons and dielectric properties. *Phys. Rev. Lett.* **48**, 406–409 (1982).

27. Monserrat, B., Drummond, N. D. & Needs, R. J. Anharmonic vibrational properties in periodic systems: energy, electron–phonon coupling, and stress. *Phys. Rev. B* **87**, 144302 (2013).

28. Lee, C., Yang, W. & Parr, R. G. Development of the Colle–Salvetti correlation-energy formula into a functional of the electron density. *Phys. Rev. B* **37**, 785–789 (1988).

29. Becke, A. D. Density-functional exchange–energy approximation with correct asymptotic behavior. *Phys. Rev. A* **38**, 3098–3100 (1988).
